# Using food-web theory to conserve ecosystems

**DOI:** 10.1038/ncomms10245

**Published:** 2016-01-18

**Authors:** E. McDonald-Madden, R. Sabbadin, E. T. Game, P. W. J. Baxter, I. Chadès, H. P. Possingham

**Affiliations:** 1 School of Geography, Planning and Environmental Management, University of Queensland, St Lucia, Queensland 4072, Australia; 2 Unité de Mathématiques et Informatique Appliquées, Toulouse, INRA UR 875, BP 27 F-31326 Castanet-Tolosan, France; 3 The Nature Conservancy, Conservation Science, South Brisbane, Queensland 4101, Australia; 4 Centre for Applications in Natural Resource Mathematics, School of Mathematics and Physics, The University of Queensland, St Lucia, Queensland 4072, Australia; 5 CSIRO, Ecosciences Precinct, Dutton Park, Queensland 4102, Australia; 6 School of Biological Sciences, University of Queensland, St Lucia, Queensland 4072, Australia; 7 School of Mathematics and Physics, The University of Queensland, St Lucia, Queensland 4072, Australia

## Abstract

Food-web theory can be a powerful guide to the management of complex ecosystems. However, we show that indices of species importance common in food-web and network theory can be a poor guide to ecosystem management, resulting in significantly more extinctions than necessary. We use Bayesian Networks and Constrained Combinatorial Optimization to find optimal management strategies for a wide range of real and hypothetical food webs. This Artificial Intelligence approach provides the ability to test the performance of any index for prioritizing species management in a network. While no single network theory index provides an appropriate guide to management for all food webs, a modified version of the Google PageRank algorithm reliably minimizes the chance and severity of negative outcomes. Our analysis shows that by prioritizing ecosystem management based on the network-wide impact of species protection rather than species loss, we can substantially improve conservation outcomes.

## Abstract (web summary)

The influence of species conservation on food webs is less well understood than the effects of species loss. Here, the authors test several indices against optimal food web management and find no current metrics are reliably effective at identifying species conservation priorities.

## Graphical abstract

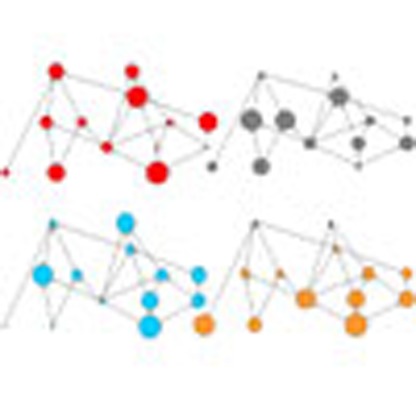

Food webs describe the trophic links between producers and consumers, and consumers and their predators, and are the most widely recognized representation of the interactions between species in an ecosystem^1^,^2^. Food web research has increasingly focused on what food webs can reveal about the vulnerability of ecosystems to species loss. Numerous studies have investigated the cascading impacts of removing a species from a food web and thus the ‘robustness' of different food-web structures to species extinction^3^,^4^,^5^,^6^. This work has been extended to identify the characteristics of species (for example, their position in the food web) that, if removed, accelerate ecosystem collapse^7^,^8^,^9^, and also species that, if removed, might limit further extinction cascades^10^. The most obvious interpretation of these results is that those species on which the robustness of a food web is most dependent should be the highest priority for management.

A subtle but important difference exists between investigating the impact of species removal and species protection. We know from empirical evidence that even when species are actively managed they can still be lost (for example, ref. 11), and lack of management does not necessarily condemn species to extinction; thus, the benefits of managing a species in a food web are not strictly equivalent to the consequence of its removal. Without investigating how the benefits of management (rather than the consequences of removal) flow through a food web, one cannot necessarily make conclusions about the importance of conserving a particular species. How food webs should be used to identify the best combination of species to manage remains an open and pressing question.

The scale of the global biodiversity crisis means that current investment in conservation is inadequate to conserve all species^12^ so prudent allocation of effort is essential. Decisions about the allocation of conservation resources are often made with a focus on individual species (for example, ref. 13). The management of any one species is, however, likely to impact other species in an ecosystem. Considering individual species in isolation when making conservation management decisions may be detrimental to other, non-focal species in the system, but also ultimately to the very species we are aiming to protect if we are unable to sustain critical ecosystem links^14^. The potential of food webs to guide ecosystem management and conservation is widely recognized^14^,^15^,^16^,^17^, and ecosystem models (akin to food webs) are frequently used to simulate the ecosystem impact of alternative fisheries management actions (for example, refs 18, 19). Here we demonstrate how food webs can be used to consider the net effects of management options across whole ecosystems, and with this ability, identify the optimal subset of species to manage to reach a system-wide objective.

To use food webs in deriving optimal strategies for the management of multiple species, we must incorporate the links between species, the threats to species survival, and the propagation of these threats throughout the system. Food-web studies have conventionally treated species persistence either through a ‘topological approach' where extinctions are determined solely by web structure (for example, (refs 4, 20, 21)) or by a dynamic approach where species interactions are represented by energy flow models (for example, ref. 5). The topological approach fails to represent the diversity in interaction strengths between species^22^. In contrast, dynamic approaches are complex, challenging to parameterize and require significant computational resources^22^. Eklöf *et al*.^22^ showed that Bayesian Belief Networks (BBNs) can capture the majority of secondary extinctions forecast by dynamic food web (for example, ref. 23), and while they do not capture all the dynamics of a food web, can provide a computationally efficient and flexible tool for predicting secondary extinctions. We use a BBN framework to model food webs but extend it by adding management actions so as to investigate the propagation of management benefits through these webs.

We use a constrained combinatorial optimization to find the best management action for a food web given the cost of species management and a fixed budget. We investigate an objective to maximize the number of species persisting in the system. Knowing the optimal strategies for managing food webs enables us to test the management performance of indices commonly used in food-web theory to identify important species for network persistence. Numerous indices have been proposed for identifying species of importance to food web stability (for example, refs 3, 8, 20, 24). In addition to food-web-specific indices, such as keystone species, we test a range of indices originating in social network theory, and an index based on Google's PageRank algorithm. We also test the performance of a cost-effectiveness approach to prioritizing management. The set of indices and approaches we test differ in the degree of food-web complexity (presence of links between species, trophic structure of the food web, and interaction strengths between species) that they consider when prioritizing species management. The size and structure of food webs have been linked to their robustness to species removals^4^,^5^,^24^. Therefore, to ensure the generality of our results, we derive the optimal strategy and compare the set of management indices across six real food webs ranging in size and connectivity and for 40 hypothetical food webs of differing connectivity.

We find that no single network theory index provides a robust guide to the management of food webs. The performance of these metrics varies widely with the specific characteristics of the system. A modified version of the Google PageRank algorithm, however, reliably minimizes the chance and severity of negative outcomes and may prove a reasonable metric to guide risk-adverse managers. We show that prioritizing ecosystem management based on the network-wide impact of species protection rather than species loss, could lead to substantially improved conservation outcomes.

## Results

### A greedy heuristic for optimal management

**Figure 1:**
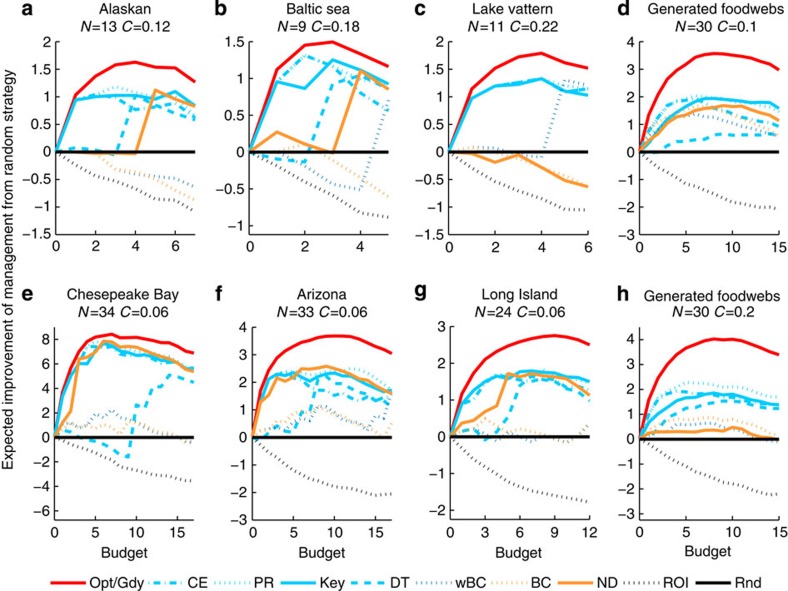
Expected performance of food web management based on each index/approach compared to a random strategy. Performance is the number of species surviving and is averaged across 100 simulations of extinction risk (drawn from a Beta distribution with *α*=2 and *β*=8) and interaction strengths (drawn from a lognormal distribution with log-mean—3.0 and log-s.d.—1.5) for six real food webs (**a**–**c**,**e**–**g**) , and 10 iterations for 20 hypothetical food webs with a connectance of 0.1 (**d**) and a connectance of 0.2 (**h**). Management reduced extinction risk of a species to zero. Note, for food webs with *n*<14, the performance of the optimal approach is identical to the greedy. Black, naive; orange, interactions; dark blue, interactions and strengths; light blue, interactions and trophic structure; red, all complexities. BC, Betweenness Centrality; CE, Cascading Extinction; DT, Dominator Tree; Gdy, Greedy approach; Key, Keystone Index; ND, Node Degree; Opt, Optimal approach; PR, modified Google PageRank; Rnd, Random strategy; ROI, Return-On-Investment; wBC, weighted BC. * note for clarity not all indices are represented.

For all real and hypothetical food webs tested here, managing species on the basis of common food web indices results in more extinctions than using an optimal multispecies management strategy (Fig. 1). For large, complex food webs (>20 nodes), it proved computationally prohibitive to find the optimal management strategy. However, for food webs where both the optimal solution and the greedy heuristic were calculated, the performance of the greedy heuristic was indistinguishable from the performance of the optimal solution (Fig. 1). This result suggests that our greedy heuristic approach represents an efficient approximation of optimal food-web management, and so is referred to as such here.

### Comparison of management metrics to optimal performance

Across the food web indices tested here, the maximum potential departure from the optimal management performance ranged from 8.6% fewer species surviving when using the modified Google PageRank index, through to 60.8% fewer species when managing based on Return-On-Investment. Between these, the percentage reduction in surviving species for the other indices were; 10.7% for Cascading Extinction, 10.8% for Keystone Index, 13.8% for Bottom–up prioritization, 28.4% for Closeness Centrality, 28.4% for Node Degree, 37.8% for weighted Betweenness Centrality, 40.5% for Betweenness Centrality, 46.3% for a Random strategy, 52.2% for Dominator Tree, and 55.7% for weighted Closeness Centrality. The degree of the potential departure from optimal for all the indices was clustered based on the degree of food web complexity incorporated into the index. Although none of the food web indices were able to approximate the optimal management of a food web, the relative extent of sub-optimality in performance varied depending on the food web being managed, the budget available for management, and the degree of food-web complexity incorporated into the index (Fig. 1). In general, the more complexity incorporated into the index the better the index performed (Fig. 1) and the more robust the index to variability in interaction strengths and food web structure (Supplementary Fig. 2). A few exceptions to this general outcome are observed. When the connectance of webs was low (*C*≤0.1), Node Degree, which only incorporates information on the presence of interactions, performed comparably to those indices that incorporate information on trophic interactions (blue lines in Fig. 1). However, when the complexity of webs increased, the performance of Node Degree declined (see Fig. 1). Interestingly, weighted versions of the common centrality measures tested here, weighted betweenness Centrality and weighted Closeness Centrality, did not perform any better than versions of these measures that do not incorporate interaction strength, and in many cases performed worse. Optimal management typically yielded the greatest improvement over the other indices when the budget allowed for the management of between a quarter and a half of the species in the food web. Despite conserving fewer species than optimal management, the food-web indices, with the notable exception of return-on-investment, represented a significant improvement over randomly choosing species to manage. The return-on-investment index always performed worse than the other indices and also worse than random.

### Robustness of metric rank depending on network complexity

**Figure 2:**
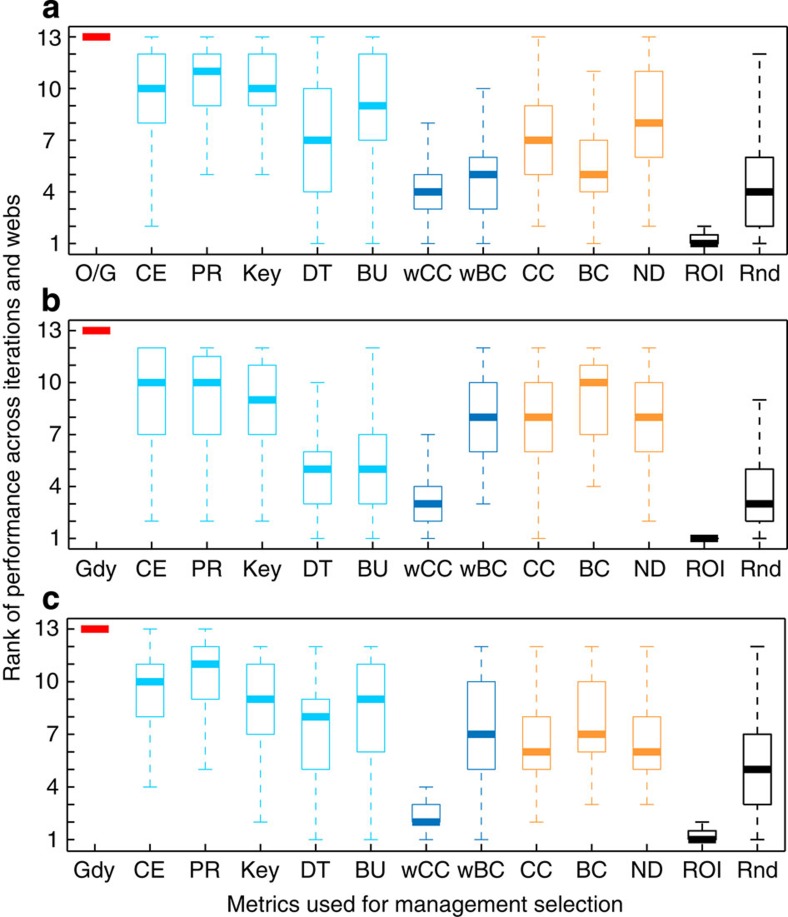
Rank of management performance given a budget of 25% required to manage all species in the food web. Rank is across 100 simulations of extinction risk (drawn from a Beta distributionwith *α*=2 and *β*=8) and interaction strengths (drawn from a lognormal distribution with log-mean—3.0 and log-s.d.—1.5) for six real webs (**a**), and across all 10 iterations for 20 hypothetical food webs with connectance of 0.1 (**b**) and 0.2 (**c**). Extinction risk with management is zero. The *greedy approach* is used for large food webs (*n*>14) instead of the optimal strategy. Standard competition ranking is used and ten is the best ranking. The center value is the median, the edges of the box the 25th and 75th percentiles, and the whiskers represent +/−1.5 the inter-quartile range. Black, naïve; orange, interactions; dark blue, interactions and strengths; light blue, interactions and trophic structure; red, all complexities. BC, Betweenness Centrality; BU, Bottom-Up Prioritization; CE, Cascading Extinction; DT, Dominator Tree; Gdy, Greedy approach; Key, Keystone Index; ND, Node Degree; O/G, Optimal or Greedy; PR, modified Google PageRank; Rnd, Random strategy; ROI, Return-On-Investment; wBC, weighted BC; wCC, weighted CC.

The management performance rank of the different indices was explored by looking at multiple management simulations for a budget fixed at 25% of that needed to manage all species in the food web (Fig. 2). Between the performance extremes of the optimal approach and the return-on-investment approach there was considerable overlap in performance across the indices (Fig. 2). However, the index with the highest median performance rank across all real and hypothetical food webs was the modified PageRank. The modified PageRank index and the Keystone Index both had the highest minimum rank across the empirical food webs and the generated food webs with connectance of 0.1 (Fig. 2), but the modified PageRank also had the least-worst outcome for generated food webs with connectance of 0.2. The modified PageRank also consistently delivered the highest potential outcome after the optimal approach.

The patterns in relative management performance of different food-web indices described above are robust to variability in the assumed interaction strength between species, food web structure, and total management budget. Supplementary Figures 3 and 4 show that the relative performance pattern remains consistent even if we vary the cost of managing species in the network, reduce the assumed effectiveness of management interventions or decrease the likelihood of high extinction probabilities. However, as management effectiveness decreases or we assume a different Beta distribution where species are more likely to have a lower chance of extinction, then the difference between optimal management and management using the other indices also decreases. Variability in the cost of managing species only served to further decrease the performance of the naïve return-on-investment approach relative to a random allocation of management efforts (Supplementary Fig. 3).

### Investigating management priorities for the Alaskan food web

**Figure 3:**
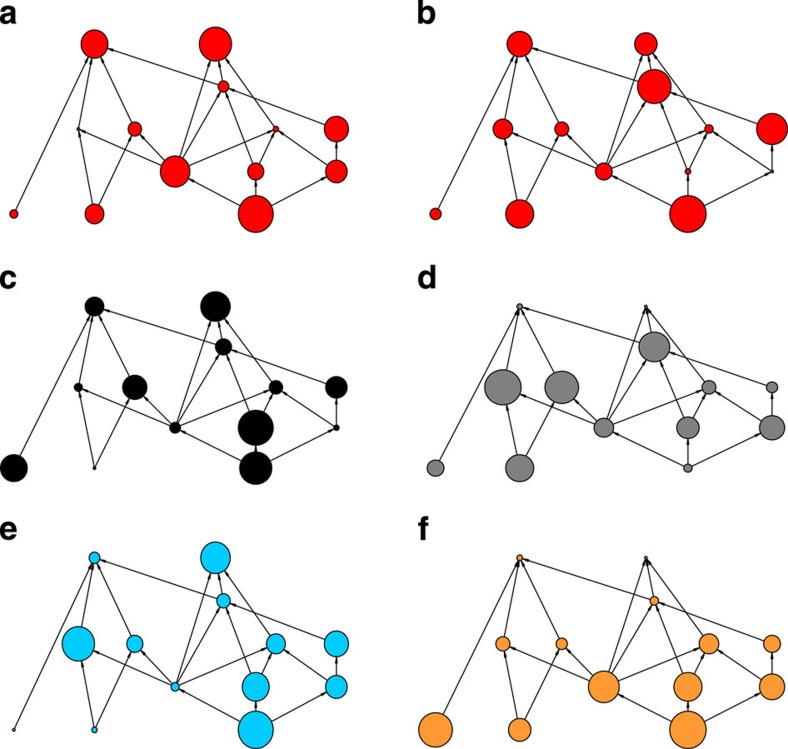
Priorities for managing the Alaskan food web using different indices/approaches. Optimal A (**a**) and B (**b**) show the optimal prioritization of species for two different assignments of species extinction risk and strength of interaction between species, where species extinction risk (drawn from a Beta distribution with *α*=2 and *β*=8) and strength of interaction between species (drawn from a lognormal distribution with log-mean—3.0 and log-s.d.—1.5. Optimal A (**a**), Return-On-Investment (**c**), Betweenness Centrality (**d**), Keystone Index (**e**), and Cascading Extinction (**f**) show the prioritization under each of these approaches for the same assignments of species extinction risk and strength of interaction between species. Prioritization rank is indicated by node radius; larger radius equals higher priority.

Although the superiority of the optimal and greedy approaches is robust to the assumed interaction strength between species, by looking in detail at a single food web, in this case the Alaskan food web, we observe that the species prioritized for management change substantially depending on the strength of interactions between species (Fig. 3a,b). Figure 3c–f also illustrates how, for the Alaskan food web, even when interaction strengths are held constant, the different social network, food web and conservation indices prioritize the management of markedly different suites of species. A detailed investigation of the species selected for management of the Alaskan food web highlights further how the species prioritized for management differ between indices (Supplementary Fig. 5). The majority of indices we investigate are deterministic and therefore select the same species to manage irrespective of interaction strengths (illustrated by the solid and white tones in Supplementary Fig. 5). The optimal and greedy approaches are able to incorporate information about interaction strengths and management, and adjust the species selected to manage accordingly (illustrated by the shaded tones in Supplementary Fig. 5).

### The impact of trophic level on management prioritization

**Figure 4:**
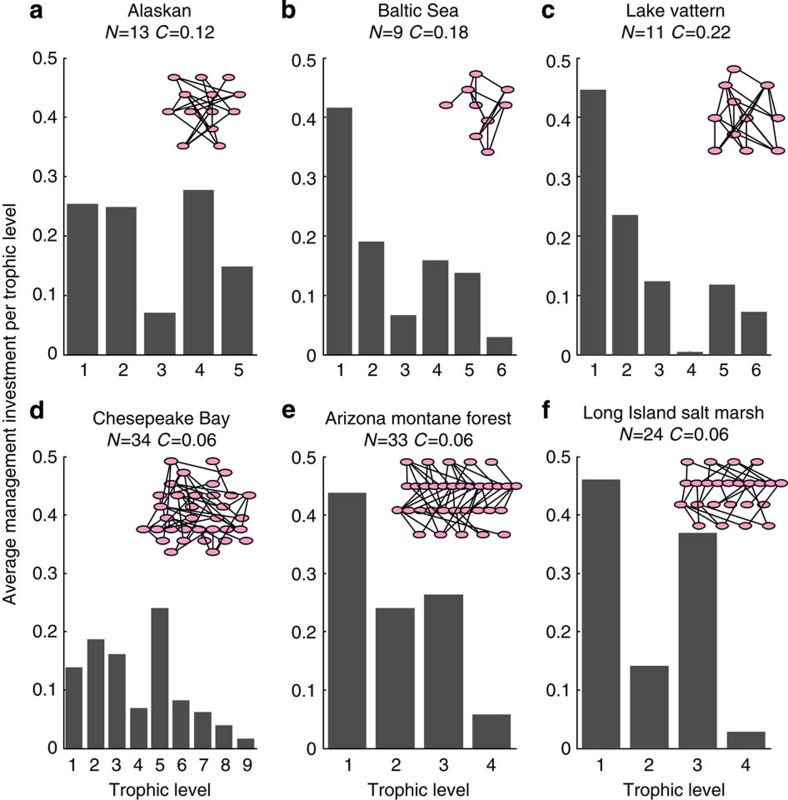
Pattern of trophic level management from optimal or greedy approach for six real food webs. For a budget set at 25% of the total budget required to manage all species in the food web, the proportion managed at each trophic level from 100 simulations of species extinction risk (drawn from a Beta distribution with *α*=2 and *β*=8) and strength of interaction between species (drawn from a lognormal distribution with log-mean—3.0 and log-s.d. 1.5). Management reduced extinction risk of a species to zero. Alaskan (**a**), Baltic Sea (**b**), Lake Vattern (**c**), Chesapeake Bay (**d**), Arizona Montane Forest (**e**), and Long Island Salt Marsh (**f**). Inset shows the structure of the real food web and illustrates the number of trophic levels and species per trophic level, note some links have been removed from larger webs for visual clarity.

Across the six real food webs explored here, there was no discernible pattern in the trophic level targeted for management by the optimal and greedy approaches (Fig. 4), and this result held regardless of variation in costs, management effectiveness or the assumed Beta distribution of extinction risk (Supplementary Fig. 6).

## Discussion

We discovered that common indices of species importance in food webs do not reveal the best species to manage to conserve the maximum number of species in those webs. The failure of management guided by food-web indices to save as many species as possible can be explained by the difference between accounting for the probabilistic impact of management on the food web, versus propagating the effects of Jenga-style removal of species. Species are neither secure with management, nor doomed without it. Instead, investing in management of a species affects the probability of that species' extinction, which in turn impacts the chance of extinction for other species in the network. The net effects on overall species persistence from not managing a species are not well approximated through the removal of that species from a food web. We demonstrate that a straightforward greedy heuristic is an excellent approximation of optimal food-web management, providing a computationally efficient way of identifying the important species to manage in large food webs. As such, the greedy approach used here represents an important addition to the indices used to evaluate food webs, and networks with probabilistic characteristics more generally.

The importance of understanding the network consequences of managing species is underscored by our finding that a single species return-on-investment approach (for example, ref. 25), a common conservation approach to prioritizing species management but one that is naïve with regard to network properties, performed substantially worse than all food-web indices. This indicates that the improvement in species survival as a result of considering which species would benefit most from management, are substantially outweighed by missed opportunities to manage the entire food web in a more holistic manner. In general, prioritization indices that neglect food-web structure perform relatively poorly, and their performance is also less robust to uncertainty in web structure and interaction strength (Supplementary Fig. 2).

Measures of the importance of a species in food webs based on aspects of network centrality have been categorized into those that describe the local interactions of a species (Local measures, for example, Node Degree and Betweeness Centrality), those that attempt to describe the species position within the entire community (Global network measures—for example, Closeness Centrality and PageRank), and those that take an intermediate approach and measure importance at a ‘meso-scale' (for example, Keystone Index). It has been proposed that mesoscale approaches, those that incorporate the interactions across the entire network but reduce their importance with distance from a species, could provide a useful tool for conservation^7^,^26^. Although in our results a mesoscale measure (Keystone Index) performs well for webs with relatively low connectance, index performance did not seem to relate to a global, meso, or local taxonomy in a consistent manner. Node Degree, a local centrality measure, also performed relatively well for low connectance webs, while the PageRank approach, akin to a global centrality measure, had the highest median performance across real and hypothetical food webs.

Previous food-web research has posed numerous indices for finding important species in food webs; from different centrality measures (as mentioned above) to the identification of species that form ‘energy bottlenecks' see (ref. 27). In our work, no single conventional food-web index provided a reliable guide to the optimal management of all food webs. From a management decision perspective, what is perhaps more interesting than the relative optimality of the median performance of the indices, is how badly an index might do in terms of species protection given the underlying uncertainty in food web parameters. The PageRank index serves not only as a consistently high performer across the indices but also serves as a sort of ‘mini-max' strategy (see ref. 28), guaranteeing the least-worst performance of all the indices. As such, the PageRank approach could offer decision-makers a way of avoiding the worst outcome when an optimal approach cannot be used.

To avoid the complex parameterization required for fully dynamic food-web models Eklöf *et al*.^22^ used BBNs to represent food webs. They show that many of the secondary extinctions in dynamic food-web models are captured by the BBN. Our model can be seen as an extension of Eklöf *et al*.^22^, using BBNs to consider both prey species importance and conservation management actions. No previous study has investigated optimal management in food webs, a mathematically and computationally challenging task. Our approach evaluates all possible permutations of species to manage. For a 30-node food web where half the species could be managed this equates to over 155 million management alternatives. While the size of the generated food webs we investigate are in the range of many recent dynamic studies, for example, 20 to 60 nodes^8^,^22^,^23^, many empirical webs are much larger, (for example, ref. 29) and currently beyond the computational ability of our approach. However, the application of our approach to real management will rarely involve search across all possible combinations because there are unlikely to be identified management actions for each species in a large web.

Our framework is a step towards decision support for optimally managing food webs. In building this approach, however, we have made a number of assumptions that should be evaluated in future work. In assigning initial values for interaction strengths within the food webs we analysed that we aimed for generality and derived initial probability of persistence from a Beta distribution and prey dependence from a lognormal distribution, see (ref. 6). Determining long-term conditional survival probabilities and prey dependence will always be a challenge, however, classical methods of learning BBN conditional probabilities from data on species occurrence or expert knowledge could be applied^30^. The strength of interactions between species based on energy flows have been shown to have an important impact on secondary extinctions in food webs, (for example, ref. 23). While our work does not incorporate energy flows as biomass, an approach in doing so might consider the probability of persistence of a species proportional to the total incoming energy flow from prey species. This is similar to the approach taken by Bellingeri and Bodini^29^ who translate an energy requirement threshold into an extinction threshold and showed that the higher this threshold the less robust the ecosystem to species removal. Importantly, some indices may be more or less robust to misspecification of network properties when predicting species of importance^31^ and exploration of this with a focus on protection could greatly inform management in the face of this uncertainty.

Food webs and the theory behind them can be a powerful guide to conservation and management of ecosystems, but we have discovered that the most common measures of species importance in food webs do not lead to the best management decisions. We developed a heuristic approach that provides a benchmark by which to assess the performance of food web and network indices, and a robust way to optimally prioritize management of multiple interacting species to maximize species survival for small to medium-sized food webs. The utility of this approach to managing food webs could easily be modified to address other objectives such as the preservation of food web structure^14^ or the management of ecosystem services^15^. We hope that the advances presented here help move food-web research from statements of assertion (‘this could be useful to conservation'), towards real utility for conservation decision-making (‘this is how').

## Methods

### Bayesian belief networks as a framework for food webs

We use BBN's to model food webs where the nodes represent species and the directed edges coincide with predation links. Here an interspecies interaction represents the conditional probability of a species *i* persisting given the set of species *j* on which it feeds (*F*_*i*_) all persist (*p*_*i*_(persistence*|F*_*i*_)). In our model, we define this initial probability of persisting for all species based on a Beta distribution with *α*=2 and *β*=8. This distribution implies a mean extinction probability of 20% within a 20-year management horizon, equivalent to an IUCN threat category of endangered^32^ (that is, *p*_*i*_(persistence*| F*_*i*_)*=*0.8). We also investigate a Beta distribution with a mean 20-year extinction probability of 5 % (*α*=2 and *β*=38). The conditional probability of persistence of each species decreases as sets of food items (*f*_*i*_) are removed from the full suite of food items (*F*_*i*_), influencing the availability of prey *j*: if *f*_*i*_≠ and *f*_*i*_≠*F*_*i*_, then,


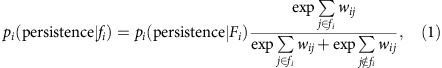


and


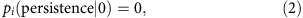


where *w*_*ij*_ is a measure of prey dependence, distributed as lognormal random variables with log-mean—3.0 and log-s.d.—1.5 as in ref. 6. The survival probabilities of basal species, which have no prey, were independent of the number of prey remaining and were hence drawn directly from the Beta distribution.

We assume that each species can be managed, or not, which will modify its 20-year conditional probability of persistence. Each management strategy is a vector, **a**, where, *a*_*i*_=1 if species *i* is managed and *a*_*i*_=0 otherwise. We assume that management is 100% effective, that is managed species with at least one prey species present (*f*_*i*_≠), cannot go extinct (that is, *p*_*i*_(persistence|*f*_*i*_)=1). We investigate the impact of management effectiveness by reducing the chance a managed species with at least one food resource can go extinct by a factor of 50% and 20%. The conditional persistence probabilities of unmanaged species are unchanged.

Good management decisions also depend on the budget available to management agencies. The total cost of a management strategy, *c*(**a**), must be less than or equal to the budget available for management (*B*):


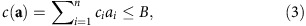


where *c*_*i*_ is the cost of managing species *i* and *n* is the total number of species.

### Optimization of conservation strategies

The equilibrium state of the food web over a 20-year period is defined as a vector **x**=(*x*_*1*_*,…, x*_*n*_), representing a group of extant species (*x*_*i*_*=*1 if species *i* is extant and *x*_*i*_*=*0 if it is extinct). To derive an optimal management strategy for a food web we define a utility based on the subset of species extant in the long term. More precisely, each extant species *i* is assigned a positive utility *U*_*i*_, and the global utility of a group of extant species is assumed to be additive:


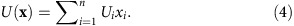


The expected value of management strategy **a**, *V*(**a**), is the expected value of the communities that persist after the implementation of management:


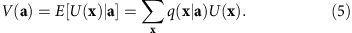


where *q*(**x***|***a**) denotes the probability that community **x** persists, given management strategy **a**. Recall that **x**=(*x*_1_*,…, x*_*n*_) is a vector of binary values, so that the sum above is taken over the 2^*n*^ possible values of **x**. Technically, *q*(**x**|**a**) is the joint probability distribution of the BBN representing the food-web trophic interactions. *V*(**a**) can be computed from the marginal probabilities *q*_*i*_(*x*_*i*_*|***a**) of the joint distribution *q*(***x***|***a***):


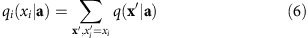


and,


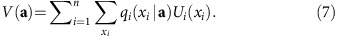


Here, the sum defining *q*_*i*_(*x*_*i*_*|***a**) is taken over all vectors **x′** varying all its components, except for *x*_*i*_, which is fixed. Therefore, the sum is taken over 2^*n*−1^ elements. See (ref. 33) for a definition of joint probability and marginal probability in a BBN.

The optimal strategy requires finding the solution a* for a fixed budget *B* and can be seen as a combinatorial optimization problem:


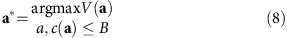


In order to compute a*, we perform an exhaustive search among all feasible management strategies **a**, looking for the strategy that maximizes *V*(**a**). We use Murphy's BNT Matlab toolbox^34^ to compute the marginal probabilities *q*_*i*_(*x*_*i*_*|***a**) of the BBN. Overall, computing an optimal strategy requires computing the marginal probabilities of as many BBNs as there are affordable management strategies. Optimization is feasible for limited budgets and relatively small numbers of species (*n*<20). For larger problems, we must turn to heuristic methods.

### Species networks management heuristics

We tested 13 heuristic methods for food-web management that incorporate varying degrees of food-web complexity, including the presence, direction and strength of interactions between species (see Table 1 for summary).

We test two approaches that are naive to the interactions between species, a Random approach and a Return-on-Investment approach calculated as the benefit of management in terms of the change in initial probability of persistence relative to the cost of managing that species^25^:


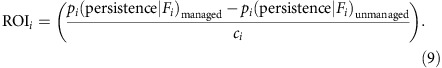


With equal management costs and effectiveness this reduces to an allocation based on the species with the highest initial probability of extinction.

We tested four indices from social network theory, Node Degree (the number of predators and prey a species has), Betweenness Centrality (how frequently a species is part of the shortest path between two species), and Closeness Centrality (the inverse of the sum of the lengths of the shortest paths from a species to all other species). Each index incorporates the presence of links only and is calculated based on (ref. 7). We also investigate a weighted version of Betweenness Centrality and Closeness Centrality^35^, where the weights are based on the measure of prey dependence, *w*_*ij*_ and represent interaction strengths.

Further, we tested three food-web-specific indices. First, the Keystone Index that assesses the importance of trophic interactions between species in the food web and the species they directly depend on, see (ref. 26). Second, a modified Google PageRank index that aims to classify species as important in the food web that support, directly or indirectly, other important species in the food web, see (ref. 36). Third, an index based on a Dominator Tree that represents the pathways essential for energy delivery throughout the food web and each species contribution to this energy deliver^27^. These approaches incorporate the presence of interactions and the trophic structure but not interaction strengths.

We also tested a topological approach to investigating the importance of species within a food web using a Cascading Extinction approach, (for example, ref. 4) to look at the impact of the independent removal of each species. Subsequent to removing one species, all species with no prey remaining were removed and this process was repeated until no more species were without resources. Species were then ranked based on the total number of species extant in the food web after these secondary extinctions ceased. No interactions strengths were considered in this approach.

Investigation of the optimal management of medium-sized food-web motifs (see Supplementary Fig. 1) showed that species were managed from the lowest trophic level upwards and within trophic levels those species with the most predators were managed first. We investigated an approach that prioritized following these rules, and is based around the notion that species depend first and foremost on the presence of their food resources (Bottom–up Prioritization). Again no interactions strengths are considered.

Computing the optimal strategy becomes infeasible as the budget and network size increase, since the number of feasible management strategies becomes too large for these to be exhaustively searched. We investigate a Greedy heuristic that instead of using exhaustive search only evaluates a small subset of possible strategies by:

1. First, it evaluates all strategies conserving a single species *i* (provided *c*_*i*_ *≤ B*). Let {*i*_*1*_} be the best such strategy.
2. Next it evaluates all pairs of species {*i*_*1*_*, i≠i*_*1*_} obtained by adding a single species to *i*_*1*_, for a total cost no more than *B*. It keeps the ‘best' such pair, {*i*_*1*_*,i*_*2*_}.
3. The greedy process of adding single species to the already selected subset, without removing previously selected species, continues until the full budget *B* is expended.

Both the Greedy and optimal approaches incorporate all three levels of complexity we consider.

### Empirical food webs to investigate management

We investigate the management of six real food webs, the Alaskan, Baltic Sea, Lake Vattern, Chesapeake Bay, Arizona Montane forest and Long Island Salt Marsh food webs. Food webs range in size from 9 species to 34 species, and in complexity with connectance value between 0.059 and 0.22 and from 4 to 9 trophic levels (see Supplementary Table 1). Data for all food webs except for the Alaskan food web see (ref. 37) were obtained from the Ecologists' Co-Operative Web Bank^38^. The food web datasets supporting this article are available at http://hdl.handle.net/10209/306.

### Generating food webs to investigate management

We constructed networks representative of ecological communities, based on the food web-niche model^39^, adjusted to prevent closed loops and cannibalism as in ref. 22. The niche model is considered the leading static food-web model as it explains the topology of a large number of empirical food webs, (for example, refs 6, 10, 40, 41) and is the base model used to generate the initial structure of food webs in many studies of dynamic food webs, (for example, refs 22, 23, 42). We generated 20 hypothetical food webs consisting of 30 species with a connectance value of 0.1, and 20 food webs consisting of 30 species with a connectance value of 0.2. This range of connectance is within the range observed for empirical food webs and is a common interval used in the study of food webs, (for example, refs 4, 6, 40).

### Testing performance of management approaches

Each management heuristic was used to make management decisions on each empirical food web and generated food web. Species in each food web were ranked based on each heuristic. This ranked list was then used to prioritize species to manage based on each heuristic. Species were managed in order until the budget, *B*, was expended. If a species was too expensive to manage given the remaining budget, then this species was skipped and the highest-ranked affordable species in the list of species was considered. For a summary of the steps we followed to assess the performance see the Supplementary Information.

Management performance of each heuristic approach was tested on each real food web with 100 iterations of initial probability of extinction and also 100 iterations with variable cost of management and initial probability of extinction. Management performance was also tested for 20 hypothetical webs of 30 nodes with 10 iterations of initial probability of extinction. First, notional costs of management were all set to one unit. The performance of each heuristic was explored under variable management costs that were drawn uniformly across non-negative integers between zero and five, that is some species cost up to five times more to manage than the cheapest species to manage.

We compared the management performance of each heuristic approach and the optimal strategy based on the expected number of species present after management, calculated as the average performance across all iterations. To investigate the consistency of performance of each heuristic across all iterations we ranked their performance in comparison with all other approaches. Ranking is based on standard competition ranking where outcomes of the same value are given an equal ranking, for example, if two heuristics both were the best ranked they would both be given a ranking of ten (for example, 10,10,9,8 and so on). To search for rules of thumb that mimic the optimal or greedy management approaches we investigated whether there was consistency in the trophic level being targeted by these approaches. The average investment in managing species in each trophic level, that is the sum of the times a species was managed across all iterations relative to the total number of species in that trophic level and normalized across all trophic levels, was used as a measure of the relative importance of the trophic level for optimal management.

## Additional information

**How to cite this article**: McDonald-Madden, E. *et al*. Using food-web theory to conserve ecosystems. *Nat. Commun.* 7:10245 doi: 10.1038/ncomms10245 (2016).

## Supplementary Material

Supplementary Information Supplementary Figures 1-6, Supplementary Table 1, Supplementary Methods and Supplementary References.

## Figures and Tables

**Table 1:** Summary of food-web complexities incorporated by each approach.

Prioritization approach*	Interaction	Trophic structure	Interaction strength	Reference
Random	—	—	—	
Return-on-Investment	—	—	—	Joseph *et al*.^25^
Node Degree	✓	—	—	Estrada *et al*.^7^
Betweenness Centrality	✓	—	—	Estrada *et al*.^7^
Closeness Centrality	✓	—	—	Estrada *et al*.^7^
Weighted Betweenness Centrality	✓	—	✓	Opsahl *et al*.^35^
Weighted Closeness Centrality	✓	—	✓	Opsahl *et al*.^35^
Dominator Tree	✓	✓	—	Allesina *et al*.^27^
Keystone Index	✓	✓	—	Jordán *et al*.^9^
Bottom–Up Prioritization	✓	✓	—	
PageRank	✓	✓	—	Allesina *et al*.^36^
Cascading Extinction	✓	✓	—	
Greedy algorithm	✓	✓	✓	
Optimal strategy	✓	✓	✓	

^*^Groupings represent classes of indices based on the degree and type of complexity incorporated.
