# Supplementary material for: Using food-web theory to conserve ecosystems
Source: Nat Commun. 2016 Jan 18;7:10245. doi: 10.1038/ncomms10245 (PMC4735605; doi:10.1038/ncomms10245)
Supplement: Supplementary Information — Supplementary Figures 1-6, Supplementary Table 1, Supplementary Methods and Supplementary References. [file ncomms10245-s1.pdf]

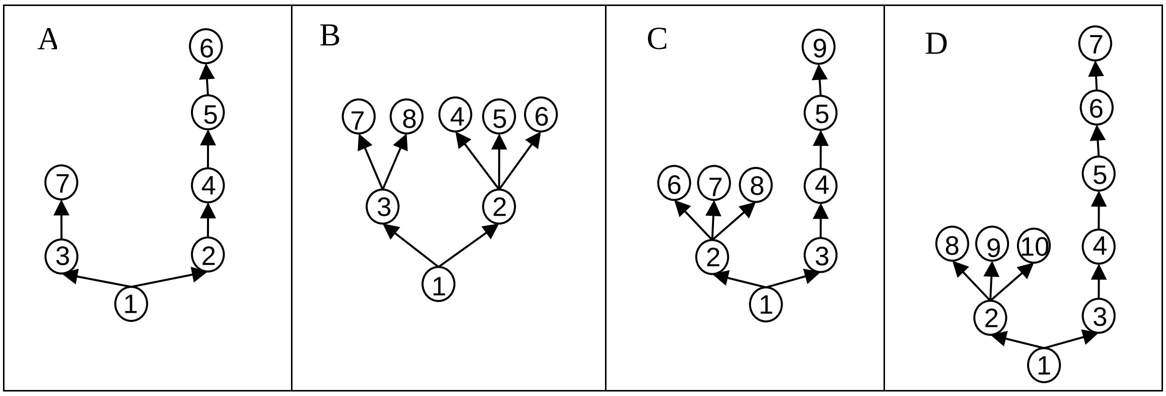

Supplementary Figure 1: Optimal management strategy for medium size motifs to investigate optimal management of food webs. Motifs are derived to understand the influence on management of A) the number of species supported within a food chain, B) the number of top predators supported by a consumer, C) tradeoff between predation impacts and the number of species support within a food chain when the same numbers of nodes are incorporated on each side of the motif, and D) the importance of predator impacts and the number of species within a food chain when more species are on one side of the motif. Numbers indicate the order of species to manage as the budget increases. Here the probability of extinction of all species and the cost of management of each species are equal. Note the order of managing top order predators does not matter under the scenario shown.

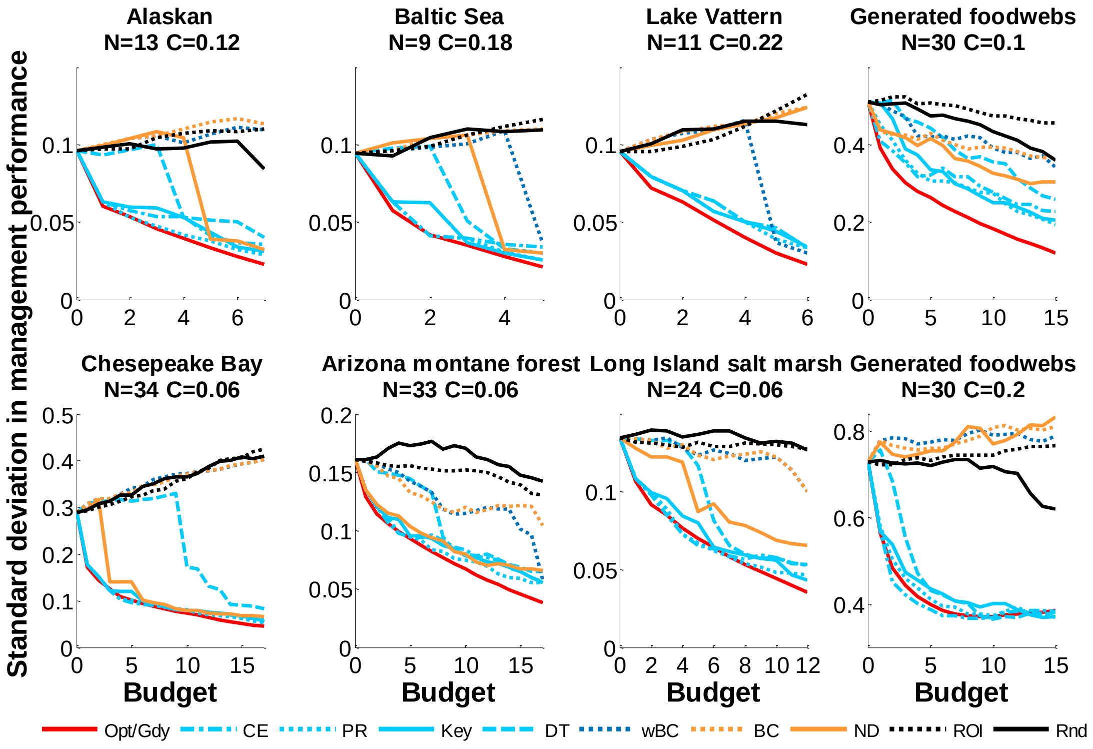

Supplementary Figure 2: Standard deviation of food web management based on each index/approach. Performance is the number of species surviving and is averaged across 100 simulations of extinction risk (drawn from a Beta distributionwith α = 2 and β = 8) and interaction strengths (drawn from a lognormal distribution with log-mean –3.0 and log-standard deviation 1.5) for six real food webs (A-C,E-G) , and ten iterations for 20 hypothetical food webs with a connectance of 0.1 (D) and a connectance of 0.2 (H). Management reduced extinction risk of a species to zero. Note, for food webs with *n*<14, the performance of the *optimal approach* is identical to the *greedy*. Colors represent degree of complexity of index/approach (see table 1). *Opt = Optimal approach, Gdy = Greedy approach, CE = Cascading extinction, PR = modified Google PageRank*^{TM}*, Key = Keystone index, DT = Dominator Tree, wBC = Weighted Betweenness Centrality, BC = Betweenness Centrality, ND = Node degree, ROI = Return-on-investment,* and *Rnd = Random strategy.* *Note for clarity not all indices are represented.

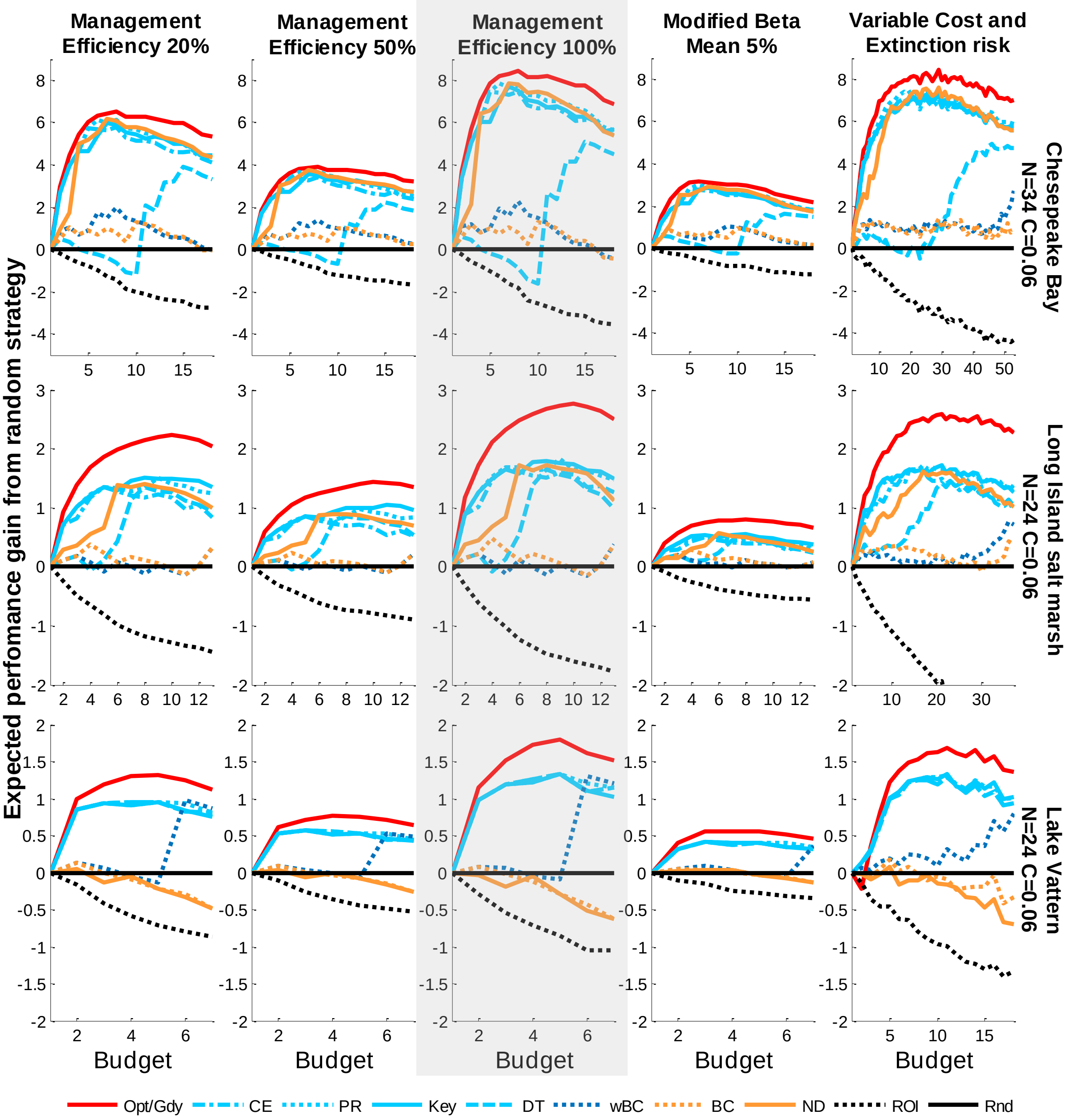

Supplementary Figure 3: Expected performance of food web management based on each index/approach compared to a random strategy. Performance is based on the number of species surviving and is averaged across 100 simulations of species extinction risk (drawn from a Beta distribution with $\alpha = 2$ and $\beta = 8$ unless stated otherwise) and interaction strengths (drawn from a lognormal distribution with log-mean –3.0 and log-standard deviation 1.5) for three real food webs, Chesepeake Bay (Row 1), Long Island Salt Marsh (Row 2) and Lake Vattern (Row 3). Results are presented when management effectiveness is reduced to 20% and management cost is equal (Column 1), when management effectiveness is 50% and cost of management is equal (Column 2), when management effectiveness is 100% and cost of management is equal (Column 3, in shaded box as based on results in main text), when management effectiveness is 100% and extinction risk is drawn from a Beta distribution ($\alpha = 2$ and $\beta = 38$)

where mean extinction is 5% (Column 4), and where management effectiveness is 100% and the cost of management and risk of extinction are varied across 100 simulations (Column 5, cost drawn from a uniform distribution between 0 and 5). Note, where computed (food webs with $n<14$), the performance of the optimal approach is identical to a *greedy* approach. Colors represent degree of complexity of index/approach (see table 1). *Opt = Optimal approach ,Gdy = Greedy approach, CE = Cascading extinction, PR = modified Google PageRank*^{TM}*, Key = Keystone index, DT = Dominator Tree, wBC = Weighted Betweenness Centrality, BC = Betweenness Centrality, ND = Node degree, ROI = Return-on-investment,* and *Rnd = Random strategy.**Note for clarity not all indices are represented.

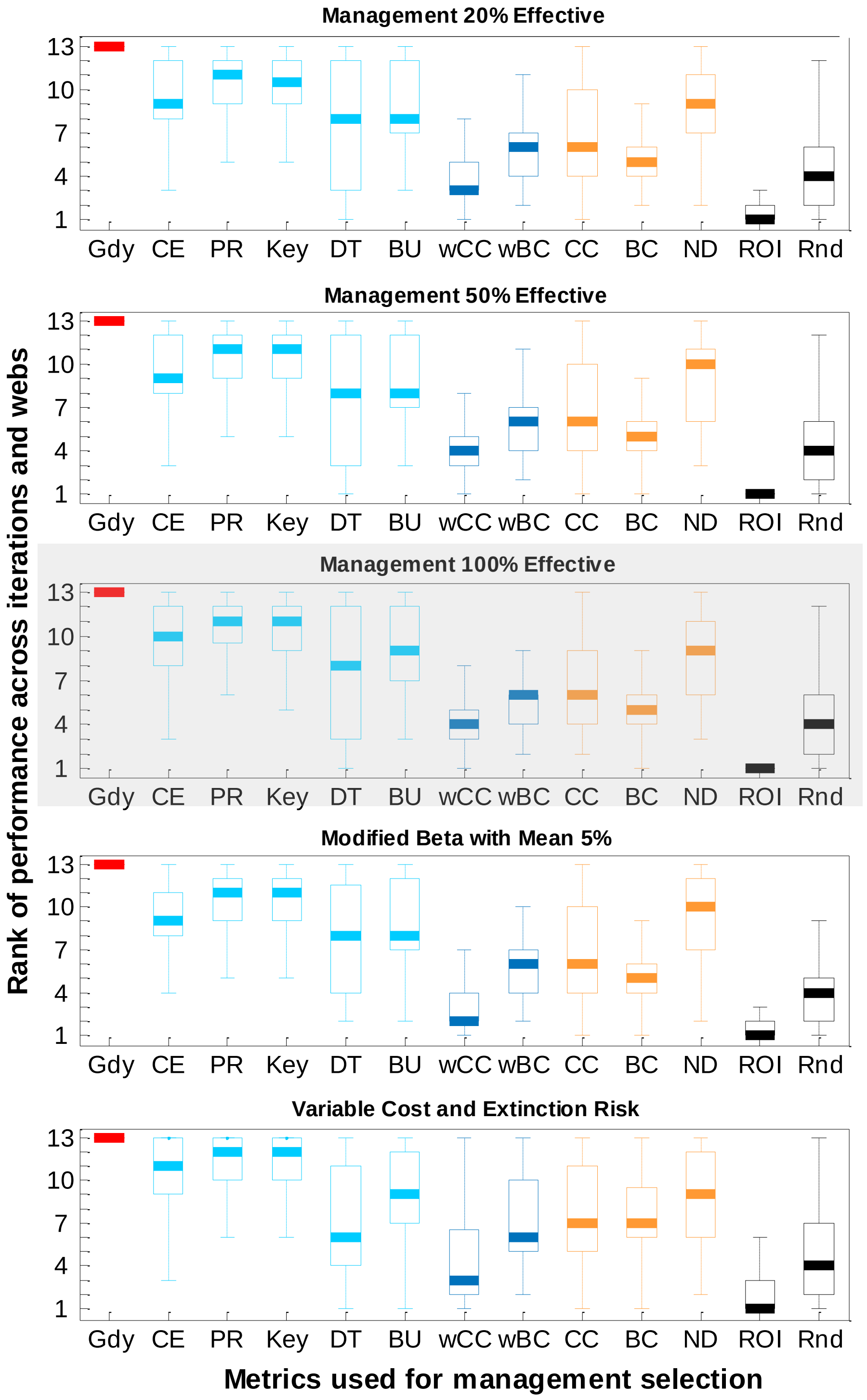

Supplementary Figure 4: Rank of management performance under limited budget. Rank order was calculated across all 100 simulations of species extinction risk (drawn from a Beta distribution with $\alpha = 2$ and $\beta = 8$ unless stated otherwise) and strength of interaction between species (drawn from a lognormal distribution with log-mean –3.0 and log-standard deviation 1.5) for three real webs (Long Island, Chesapeake Bay and Lake Vattern). Budget represented is 25% of the total budget required to manage all species in the food web. Results are presented when management effectiveness is reduced to 20% and management cost is equal (Row 1), when management effectiveness is 50% and cost of management is

equal (Row 2), when management effectiveness is 100% and cost of management is equal (Row 3, in shaded box as based on results in main text), when management effectiveness is 100% and extinction risk is drawn from a Beta distribution ($\alpha = 2$ and $\beta = 28$) where mean extinction is 5% (Row 4), and where management effectiveness is 100% and the cost of management and risk of extinction are varied across 100 simulations (Row 5, cost drawn from a uniform distribution from 0 to 5). For computational reasons, the Greedy approach is used for large food webs (n>14) instead of the optimal strategy. Colors represent degree of complexity of index/approach (see table 1). Ranking is based on standard competition outcomes of the same value are given an equal ranking. Here a ranking of ten is the best ranking. The center value is the median, the edges of the box the 25th and 75th percentiles, and the whiskers represent +/- 1.5 the inter-quartile range. *Opt = Optimal approach ,Gdy = Greedy approach, CE = Cascading extinction, PR = modified Google PageRank^{TM}, Key = Keystone index, DT = Dominator Tree, BU = Bottom-up Prioritization, wCC = Weighted Closeness Centrality , wBC = Weighted Betweenness Centrality, CC = Closeness Centrality , BC = Betweenness Centrality, ND = Node degree, ROI = Return-on-investment*, and *Rnd = Random strategy.*

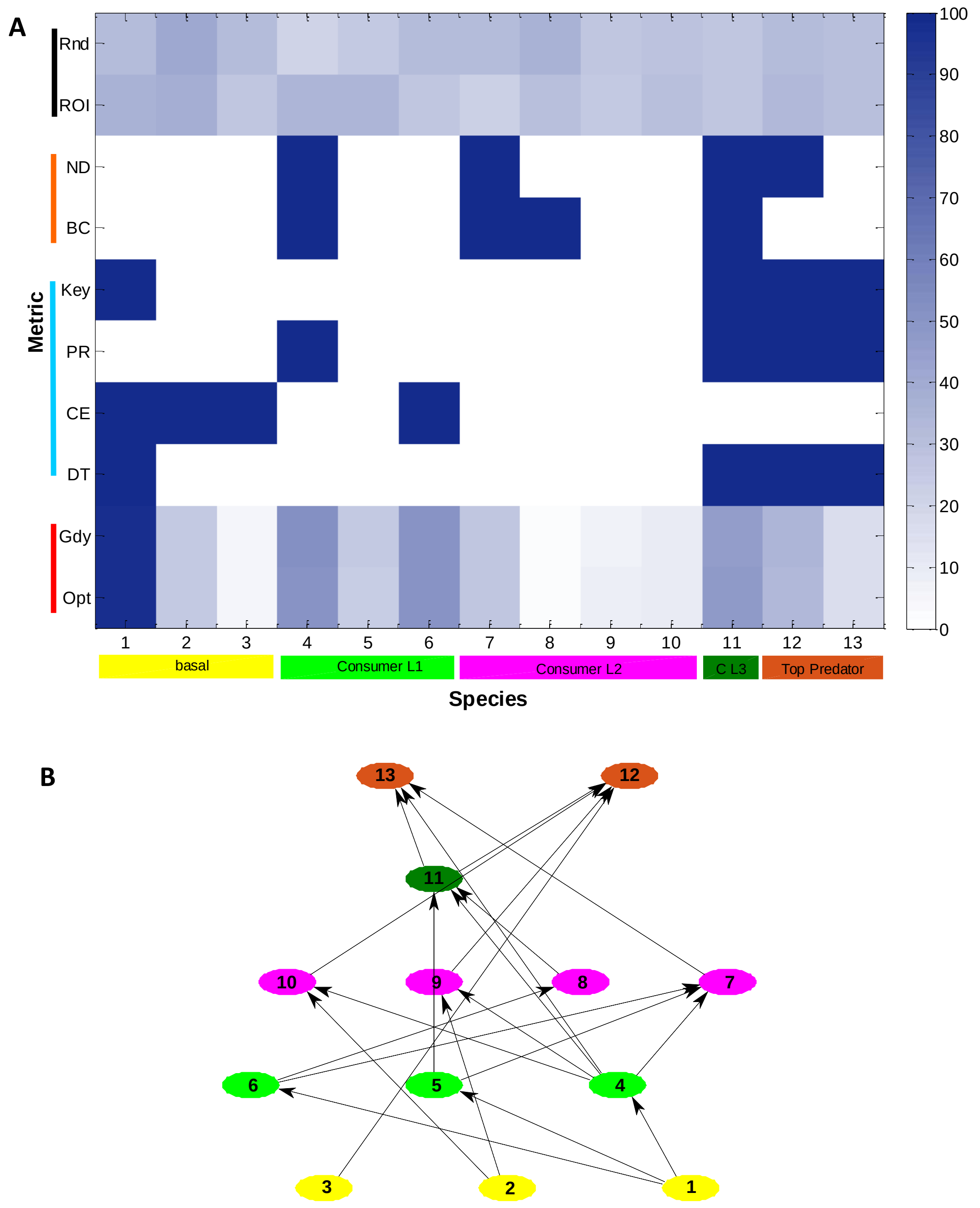

Supplementary Figure 5: A) Percentage of iterations each species in the Alaskan food web is selected for management. Dark blue are selected most often and white are never selected (see colorbar). Trophic level is shown in colors below x-axis and approach complexity in colors next to y-axis. Results are for a budget set at 25% of the total budget required to manage all species in the food web, and based on 100 simulations of species extinction risk (drawn from a Beta distribution with $\alpha = 2$ and $\beta = 8$), and strength of interaction between species (drawn from a lognormal distribution with log-mean –3.0 and log-standard deviation 1.5). Management effectiveness is 100% and cost of management is equal. B) **Shows the structure of the Alaskan food web**, colors represent trophic levels as in A.

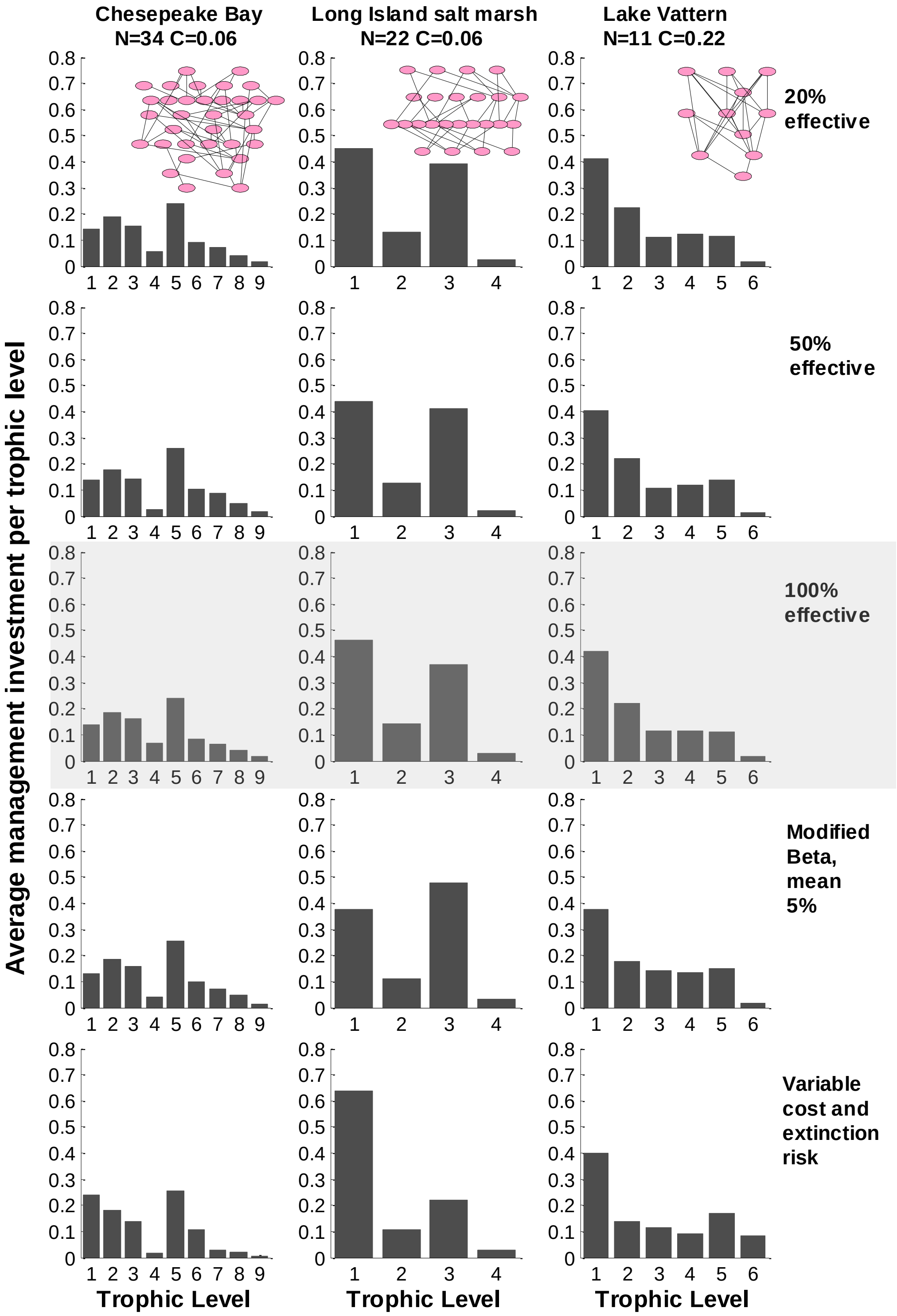

Supplementary Figure 6: Pattern of trophic level management from optimal or *greedy* approach for three real food webs. For a budget set at 25% of the total budget required to manage all species in the food web, the proportion managed at each trophic level from 100 simulations of species extinction risk (drawn from a Beta distribution with α = 2 and β = 8 unless stated otherwise), and strength of interaction between species (drawn from a lognormal distribution with log-mean –3.0 and log-standard deviation 1.5). Results are presented when management effectiveness is reduced to 20% and management cost is equal (Row 1), when management effectiveness is 50% and cost of management is equal (Row 2), when management

effectiveness is 100% and cost of management is equal (Row 3, in shaded box as based on results in main text), when management effectiveness is 100% and extinction risk is drawn from a Beta distribution ($\alpha = 2$ and $\beta = 38$) where mean extinction is 5% (Row 4), and where management effectiveness is 100% and the cost of management and risk of extinction are varied across 100 simulations (Row 5, cost drawn from a uniform distribution between 0 and 5). Inset shows the structure of the real food web and illustrates the number of trophic levels and species per trophic level, note some links have been removed from larger webs for visual clarity.

Supplementary Table 1: Summary of real food webs

Web Name	Species	Links	Connect-Ance	Trophic levels	Ecosystem	Reference
**Alaskan**	13	21	0.1243	5	Inshore Marine	[1]
**Baltic Sea**	9	15	0.1852	6	Marine pelagic	[2]*
**Lake Vittern**	11	27	0.2231	6	Freshwater pelagic	[2]*
**Chesapeake Bay**	34	75	0.0649	9	Estuary	[3]*
**Arizona Montane forest**	33	69	0.0634	4	Montane forest	[4]*
**Long Island Salt Marsh**	24	34	0.0590	4	Salt marsh	[5]*

** Data obtained from the Ecologists' Co-Operative Web Bank [6] .*

SupplementaryMethods:

Method sequence for assessing performance of indices

1. Generate web or select empirical web
    a. Use Niche model to generate 40 webs with 30 nodes and connectance of 0.1 and 0.2
2. Generate parameters
    a. probabilities of extinction using Beta distribution
    b. Interaction strengths using Log Normal distribution
    c. Costs using uniform distribution or equal
3. Combine Probability of extinction and Interaction strength using equation 1 and Generate CPT tables
4. Select budget available
5. Best strategies from each index
    a. Calculate index for each node
    b. Rank index from high to low
    c. Assign cost to each node from 2)
    d. Choose down the list of nodes rank in b) until entire budget expended
        i. Skip nodes if too expensive for remaining budget and continue down list

e. Evaluate final policy using BBN
f. Store policy and Expected Number of species surviving from e)

6. Generate optimal policy; store it and Expected Number of species surviving from it.
7. Generate Greedy Heuristic
   a. Evaluating all strategies conserving a single species *i* (provided $c_i \le B$). Let $\{i_1\}$ be the best such strategy.
   b. Next it evaluates all pairs of species $\{i_1, i \neq i_1\}$ obtained by adding a single species to i_1, for a total cost no more than *B*. It keeps the "best" such pair, $\{i_1, i_2\}$.
   c. The *greedy* process of adding single species to the already selected subset, without removing previously selected species, continues until the full budget *B* is expended.
   d. Store greedy strategy and Expected Number of species surviving
8. Repeat 2 to 7 for each budget up to half the budget required to manage all nodes
9. Repeat 1 to 7 for all webs both generated and empirical

Supplementary References

1 Estes, J. A., Doak, D. F., Springer, A. M. & Williams, T. M. Causes and consequences of marine mammal population declines in southwest Alaska: a food-web perspective. *Philosophical Transactions of the Royal Society B-Biological Sciences* **364**, 1647-1658, doi:10.1098/rstb.2008.0231 (2009).

2 Berg, S., Christianou, M., Jonsson, T. & Ebenman, B. Using sensitivity analysis to identify keystone species and keystone links in size-based food webs. *Oikos* **120**, 510-519, doi:10.1111/j.1600-0706.2010.18864.x (2011).

3 Baird, D. & Ulanowicz, R. E. The seasonal dynamics of the Chesapeake Bay ecosystem. *Ecol. Monogr.* **59**, 329-364 (1989).

4 Woodwell, G. M. Toxic substances and ecological cycles. *Scientifc American* **216**, 24-31 (1967).

5 Rasmussen, D. I. Biotic communities of Kaibab Plateau, Arizona. *Ecol. Monogr.* **11**, 228-275 (1941).

6 Cohen, J. E. Vol. 1.1 (New York: The Rockefeller University, 2010).
